# Gender differences in career development awards in United States’ anesthesiology and surgery departments, 2006–2016

**DOI:** 10.1186/s12871-018-0561-1

**Published:** 2018-07-27

**Authors:** Lena M. Mayes, Cynthia A. Wong, Shanta Zimmer, Ana Fernandez-Bustamante, Karsten Bartels

**Affiliations:** 1 Department of Anesthesiology, University of Colorado School of Medicine, 12401 E. 17th Avenue Leprino Office Building, 7th Floor, MS B-113, Aurora, CO 80045 USA; 2 Department of Anesthesia, University of Iowa Carver College of Medicine, Iowa City, IA USA; 3 Department of Medicine, University of Colorado School of Medicine, Aurora, CO USA

**Keywords:** Grants, Funding, Career development, Gender, Diversity, Anesthesiology, Surgery

## Abstract

**Background:**

Although the status of women in anesthesiology has advanced by many measures, obtaining career development funding remains challenging. Here, we sought to compare the characteristics of funded career development awards from the National Institutes of Health (NIH) between the specialties of anesthesiology and surgery. We hypothesized that the two groups differ in percentage of faculty with awards, gender distribution among principal investigators, as well as the number of awards promoting diversity.

**Methods:**

The NIH grant-funding database RePORT was queried for career development awards for the years 2006–2016 using the filters “Anesthesiology” and “Surgery.” Grants were characterized based on the gender of the principal investigator and whether the funding opportunity announcement indicated promotion of underrepresented minorities (URM). The 2016 Association of American Medical Colleges (AAMC) report on “Distribution of U.S. Medical School Faculty by Sex and Rank” was used to adjust comparisons according to baseline gender distributions in anesthesiology and surgery departments. Cohorts were characterized using descriptive methods and compared using Chi-square or Fisher’s exact test.

**Results:**

Based on our AAMC data query, in 2016, the number of women faculty members at the instructor or assistant professor level in U.S. medical schools was 2314 (41%) for anesthesiology and 2281 (30%) for surgery. Between 2006 and 2016, there were 88 career development grants awarded to investigators in anesthesiology departments compared to 261 in surgery departments. Of the grantees in each specialty, 29 (33%) were women in anesthesiology and 72 (28%) in surgery (*P =* 0.344). Awards to promote URM were identified for two grants (2%) in anesthesiology and nine grants (3%) in surgery (*P =* 0.737). Faculty members in surgery were more likely to receive an award than in anesthesiology (*P <* 0.0001), and women were less likely to receive an award than men (*P =* 0.026).

**Conclusions:**

The major difference between US anesthesiology and surgery departments is that the number of faculty career development awards is significantly higher in surgery departments. Future efforts should aim to identify the reasons for such differences in order to inform strategies that can improve the likelihood for junior faculty members to receive career development funding.

## Background

Research performance in anesthesiology compared with other medical specialties has historically been low [1]. Accordingly, leaders in the field have called for improvement in academic development opportunities for trainees and junior faculty members [2, 3]. Indeed, professional societies such as the Foundation for Anesthesia Education and Research (FAER), the International Anesthesia Research Society (IARS), and the Anesthesia Patient Safety Foundation (APSF) have committed substantial resources to provide research funding to young investigators in the specialty of anesthesiology [4–6].

In his 2015 *Rovenstine Lecture* “*Without Science There Is Little Art in Anesthesiology*” at the American Society of Anesthesiologists’ annual meeting, Eisenach highlighted the ongoing critical importance of supporting young investigators in anesthesiology [7]. Such support may be especially important to early career women faculty members [8]. Historically, women have lagged behind men in career advancement. A 2008 assessment of the status of women in the field of academic anesthesiology in the United States highlighted the increased participation of women in many aspects of academic anesthesiology in the previous two decades [9]. Yet, the proportion of competitive research grants awarded to women had not changed. The reasons for this lack of improvement were not clear.

Consistent with work by others [10], and to permit an evaluation of another medical specialty represented at the same institutions as anesthesiology departments, we chose to compare National Institutes of Health (NIH) career development awards in anesthesiology departments to such awards in surgery departments from 2006 through 2016. We hypothesized that the two groups differ in terms of overall number of awards as well as gender distribution among principal investigators.

## Methods

The Colorado Multi-Institutional Review Board approved this study for exemption (protocol # 17–0304). There was no requirement for informed consent.

The NIH grant-funding database, NIH RePORT [11] was queried for the following career development grant categories: K01 (Mentored Research Scientist Development Award), K08 (Mentored Clinical Scientist Research Career Development Award), and K23 (Mentored Patient-Oriented Research Career Development Award). K08 and K23 awards require a clinical doctoral degree; this is not required for K01-type awards that can be pursued with a research doctoral degree. Given the hybrid nature of K99/R00 “Pathway to Independence Awards” that includes mentored and independent components, we did not include this grant category in our analysis. All awards with an active listing for the years 2006 through 2016 were included using the NIH RePORT filter for department, choosing the identifiers “Anesthesiology” and “Surgery.” Only grants with a specified department affiliation “Anesthesiology” or “Surgery” were included in this study. We determined the gender of the principal investigator by the first name and, if required, from faculty listings from departmental websites as described by others [12]. NIH funding opportunity announcements were examined to determine if they were targeted for promotion of underrepresented minorities (URM).

To adjust for the total number of faculty members in academic anesthesiology and surgery departments, respectively, data from the 2016 Association of American Medical Colleges (AAMC) report on “Distribution of U.S. Medical School Faculty by Sex and Rank” [13] were used. Participating U.S. medical schools provide information to the AAMC online or through batch uploads. Faculty members at the instructor and assistant professor ranks were assumed eligible for career development funding. Within-specialty funding rates between women and men were calculated by using the number of 2016 faculty members at the combined assistant professor and instructor level in academic anesthesiology and surgery departments.

### Statistical analysis

Funding rates and principal investigator characteristics were analyzed using descriptive statistics, including percentages and ratios. Comparisons were made by department between type of award (K01, K08, K23), gender of principal investigator (PI), and funding opportunity announcements for promotion of URM. Using the AAMC data to determine the number of eligible faculty, funding rates were compared between gender of the PI by department. Comparisons were made using the Fisher’s exact test or Chi-square test as appropriate. A *P*-value < 0.05 was considered significant. SPSS, Version 24 (IBM Corporation, Armonk, New York) was used for statistical analysis. Prism 6.0 was used for generation of graphical displays of data (GraphPad Software, Inc., La Jolla, CA).

## Results

In the time period between 2006 and 2016, we identified 88 career development grants awarded to investigators in anesthesiology departments compared to 261 in surgery departments. One hundred one grants were awarded to women and 248 grants were awarded to men. The distribution of NIH institutes awarding grants according to specialty is depicted in Table 1.

**Table 1:** Career development grants in anesthesiology and surgery departments by funding institute/agency

Institute/Agency	Anesthesiology	Surgery	Total
NHLBI	13	64	77
NIGMS	30	36	66
NIDDK	5	49	54
NCI	1	45	46
AHRQ	4	12	16
NIA	6	8	14
NINDS	10	4	14
NICHD	4	7	11
*Other*	15	36	51
All	88	261	349

*AHRQ* Agency for Healthcare Research and Quality, *NCI* National Cancer Institute, *NHLBI* National Heart, Lung, and Blood Institute, *NIA* National Institute on Aging, *NICHD* Eunice Kennedy Shriver National Institute of Child Health and Human Development, *NIDDK* National Institute of Diabetes and Digestive and Kidney Diseases, *NIGMS* National Institute of General Medical Sciences, *NINDS* National Institute of Neurological Disorders and Stroke

**Fig. 1:**
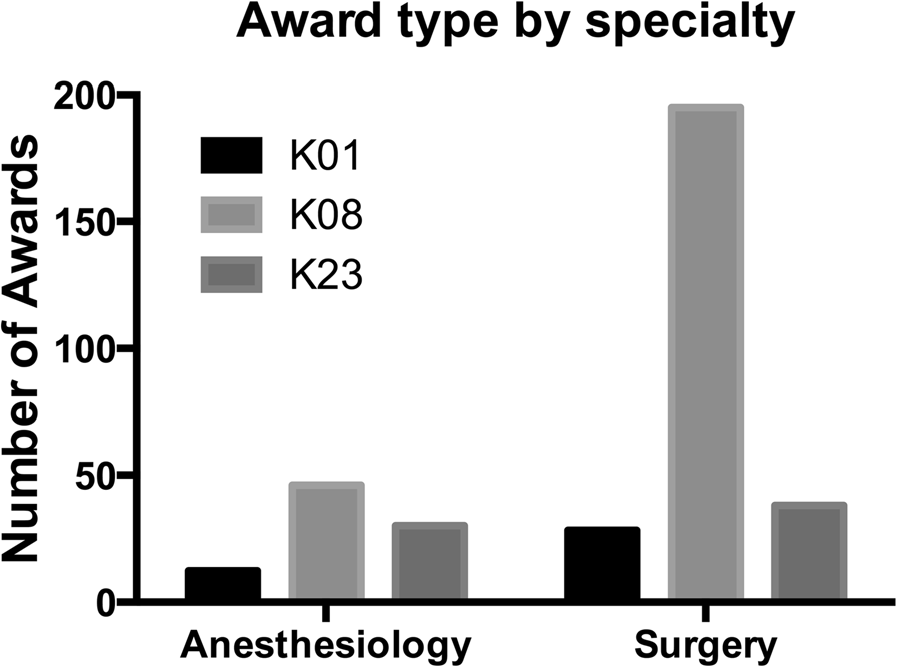
Career development awards in Anesthesiology and Surgery by funding mechanism. Comparison of award type by specialty using Chi-square test (*P =* 0.0001)

Of the identified career development awards, K08 awards were most common in both specialties. The distribution of awards differed globally by specialty (*P =* 0.0001) (Fig. 1).

**Fig. 2:**
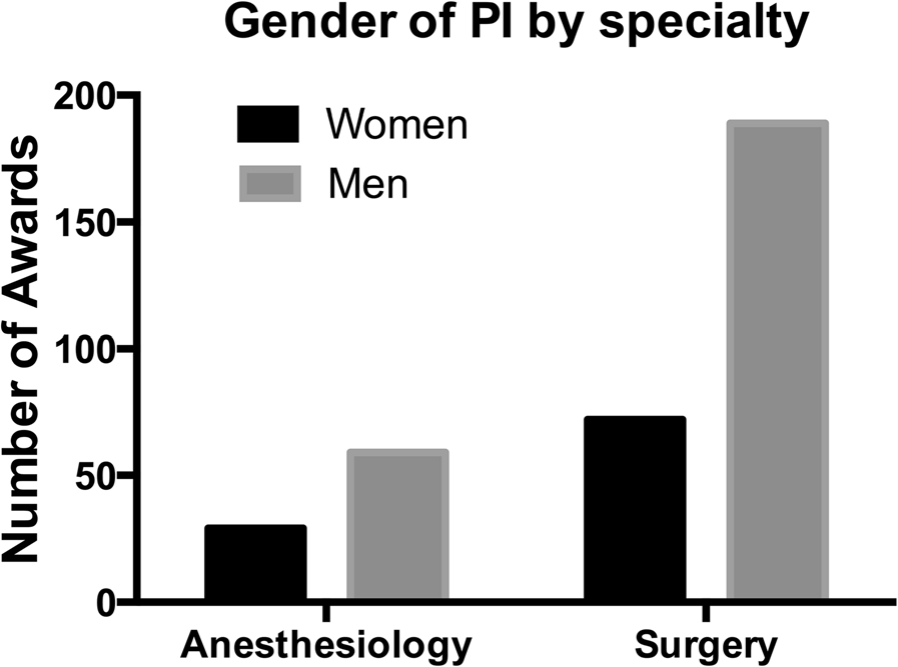
Career development awards in Anesthesiology and Surgery by gender of principal investigator (PI). There was no difference in the proportion of women grantees between the two specialties. Comparison using Chi square test (*P =* 0.344)

Of the grantees in the two specialties, 29 (33%) were women in anesthesiology and 72 (28%) in surgery (anesthesiology vs. surgery, *P =* 0.344) (Fig. 2).

Awards to promote URM were identified for two grants (2%) in the anesthesiology cohort and nine grants (3%) in the surgery cohort (*P =* 0.737, Fisher’s Exact test).

According to the 2016 AAMC report on “Distribution of U.S. Medical School Faculty by Sex and Rank,” there were 2314 (41%) women and 3387 (59%) men at the combined instructor and assistant professor rank in academic anesthesiology departments. For academic surgery departments there were 2281 (30%) women and 5325 (70%) men [13]. Overall, faculty members in surgery were more likely to receive an award than in anesthesiology (*P <* 0.0001, Chi square test). Women were less likely to receive an award than men (*P =* 0.026, Chi square test). When award rates were compared between genders within the respective specialty, the differences in award rates were not significant (anesthesiology: *P =* 0.156, surgery: *P =* 0.410, Chi square test).

## Discussion

The gross number of grants awarded to early career surgery faculty members in the past decade was almost three-fold higher than to anesthesiology faculty members. These findings need to be placed in context of the total number of early career faculty members competing for such grants in the respective specialty. When using the 2016 numbers of faculty members in the assistant professor and instructor ranks as a surrogate for grant-eligible individuals, we found that surgery faculty members were more likely to be awarded funding than anesthesiology faculty members. Although early career women faculty members in both specialties were less likely to receive grant funding than men, this finding was of only marginal statistical significance. Four additional awards towards a women PI in either specialty would have resulted in a non-significant *P-*value when comparing grant award rates by gender. Finally, funded career development grants geared specifically to promote URM faculty members were extremely uncommon in both specialties.

The relationship between gender and grant support has been examined in other specialties, often showing disadvantageous funding environments for women [14]. In a retrospective analysis of NIH grants awarded to Unites States diagnostic radiology departments, women were found to have received only 15.9% of awards and 13.3% of funding [15]. In a study reporting on 2014 NIH grants to orthopedic surgery departments, 79.5% of grants were awarded to men [16]. Although such findings may not come as a complete surprise, the finding that NIH funding rates for women physician-scientists have recently been decreasing more sharply than for men is concerning [17]. In anesthesiology, early-career grant support from the FAER has been associated with significantly higher academic productivity and subsequent NIH funding [18].

Our study adds to previous reports. Surgery faculty members are more successful in achieving NIH career development funding compared to anesthesiology faculty members. This observation appears to be primarily driven by a higher number of K08 (Mentored Clinical Scientist Research Career Development Awards) awards that often cover basic science-oriented grants submitted by applicants who must hold a clinical doctoral degree. We can only speculate that high numbers of K08-type awards in surgery departments could be due to more favorable existing departmental research infrastructure but possibly also different interests of surgery versus anesthesiology faculty members engaged in research and even in part driven by lack of anesthesiology expertise on NIH study sections. It should be noted that significant protected time for research is a core component of many surgical residencies. In a survey of 18 surgical residency programs in New England, 61% of respondents planned or had already engaged in a research elective, with the majority of residents pursuing research for 2 years or more [19]. In another survey study the University of Washington surgical residency found that 27 of 33 (82%) graduates who performed 2–3 years of research during residency were successful in obtaining NIH funding if they applied for it [20]. Also, in a 2010 analysis of career choices of 1621 MD PhD program graduates, 50 (3.1%) had chosen anesthesiology, whereas 116 (7.2%) had chosen surgery, possibly indicating a higher propensity of MD PhD graduates to choose careers in surgery [21]. When assessing combined award rates for both anesthesiology and surgery, women faculty members are only marginally less likely to be awarded funding compared to men. This finding is important since K-type funding is a critical stepping-stone to independent NIH funding [22].

Observed funding success differences between women and men following career development awards indicate less favorable long-term funding outcomes for women [23]. Culley reported 30% of anesthesiology chair persons have a history of NIH funding, compared to 62% in surgery [10]. Hence, our finding that, regardless of gender, faculty members in anesthesiology departments are less likely to obtain career development funding compared to surgery may not come as a surprise. The reasons for this finding, however, remain uncertain. We can only speculate that junior faculty members in surgery departments are receiving better support and mentoring or that the observed differences are also based on diverse baseline characteristics of men and women choosing anesthesiology versus surgery as a career.

Our study has several limitations. First, we only included funding awarded through the NIH and the Agency for Healthcare Research and Quality and not from foundations such as the FAER or IARS. However, given that we wanted to include the comparison group of surgical career development grant funding, it was necessary to choose a funding agency to which both specialties have access. Second, we could only determine gender through the principal investigator’s first name and, if required, by searching on departmental faculty webpages. While self-identified gender determination would be preferable, our approach is consistent with published approaches for gender determination in database research [12]. We did not recognize other genders than men and women in our study. Third, while we assessed the grants for funding opportunity announcements geared to faculty members with URM background, we could not ascertain ethnicity and race for individual principal investigators. Given that the assessment for URM status included only the funding opportunity announcement (NIH career development awards to promote diversity), but not the characteristics of the principle investigators or applicants, our findings do not permit any conclusions on URM status of grant applicants or awardees. Fourth, the denominator used for the gender-specific funding rates was based on AAMC data for the year 2016, not 2006–2016. This was done intentionally, as K-type career development grants usually span 3–5 years and our intent was to provide a relative, but not absolute, comparator to adjust for the difference in faculty members represented in each specialty [13]. In addition, the AAMC-based denominator does not account for the number of grant applications submitted relative to the number of grants awarded, which may differ between specialties.

## Conclusions

Based on the 2016 AAMC number of eligible faculty members, faculty members in surgery were more likely to receive an award than faculty members in anesthesiology, but the funding rates for women and men in both departments were only marginally different. Future efforts should focus on identifying the reasons for such differences in award rates to inform initiatives to boost opportunities for junior faculty members to successfully compete for NIH career development funding.

## Abbreviations

AAMC: Association of American Medical Colleges
APSF: Anesthesia Patient Safety Foundation
FAER: Foundation for Anesthesia Education and Research
IARS: International Anesthesia Research Society
NIH: National Institutes of Health
URM: Underrepresented minorities
